# Thermal Conductivity of Aluminosilicate- and Aluminum Oxide-Filled Thermosets for Injection Molding: Effect of Filler Content, Filler Size and Filler Geometry

**DOI:** 10.3390/polym10040457

**Published:** 2018-04-20

**Authors:** Yang Zhao, Zhanyu Zhai, Dietmar Drummer

**Affiliations:** Institute of Polymer Technology, University Erlangen-Nurenberg, Erlangen 91058, Germany; zhai@lkt.uni-erlangen.de (Z.Z.); drummer@lkt.uni-erlangen.de (D.D.)

**Keywords:** thermal conductive polymer composite, aluminosilicate, aluminum oxide, EMC, spheres filler, irregular filler, injection molding, Lewis-Nielsen’s equation

## Abstract

In this study, epoxy molding compounds (EMCs) with aluminosilicate (AlS) and aluminum oxide (AlO) were fabricated as fillers by a twin-screw-extruder (TSE) and shaped to plate samples using injection molding. AlS and AlO, electrical insulating mineral materials, were used as fillers to improve the thermal conductivity (λ_c_) of composites. Composites with different filler particle sizes, filler contents and filler geometry were fabricated and the influence of these variables on the λ_c_ was studied. The λ_c_ of composites was measured with the hot-disk method. The distribution of fillers in composites was observed using scanning electron microscopy (SEM). Using the Lewis-Nielsen equation, experimental values of λ_c_ were compared with those predicted. The predicted results fit the experimental values well. The result showed that λ_c_ increases significantly when the filler content of composites is approximately over 50 vol %.

## 1. Introduction

Nowadays, as the size of electronic devices decreases while their power increases, the heat transfer of electronic packaging materials has become more and more essential for long-life products. Packaging material with high thermal conductivity could improve heat transfer and hence reduce the inside temperature and thermal stress of electronic devices. Generally, dielectric liquid resins, e.g., epoxy resins, are dropped on the chip-board to form encapsulants. These protect the chip-board from mechanical damage, contaminants and moisture after curing [1]. However, pure epoxy resin normally has low thermal conductivity (0.2–0.3 W/mK) [2] which prevents the emission of heat to the surroundings and to an extent, leads to an overheating of the electronic device. To improve this aspect, different thermal conductive fillers have been introduced to the polymer matrix [3,4,5,6,7]. However, the processing methods of thermosets composites are limited. This mostly includes compression or casting molding [4,8]. Using the current processing methods, it is difficult to obtain thermal conductive composites based on thermosets with complex geometry, e.g., pin-fin heat sink. To overcome this shortcoming, a new processing method should be employed. Currently, the large series production of thermal conductive composites based on the thermoplastic matrix, especially for the components with complex geometry, has been achieved using injection molding. 

Fillers with high thermal conductivity used for thermoplastic composites, e.g., multi-wall carbon nanotubes (MWCNTs), carbon fibers, and coppers, could effectively improve the thermal conductivity of polymer composites [9,10,11,12]. While fillers with high electrical resistance such as boron nitride (BN) and aluminium nitride (AlN) have been already introduced to polymers [13,14], electrical isolation is one of the necessary properties of packaging material for those with high thermal conductivity such as electronic devices. For a significant increase in thermal conductivity, the polymer needs to be filled with a high volume. For example, to get the thermal conductivity of 1 W/mK, ca. 40 vol % BN is needed [13]. However, the applications of these materials are limited because of the high cost of raw materials. Consequently, current research activities have shifted towards the development of the thermal conductive composites ‘cost-performance’ rather than their ‘high-performance’. 

Since composites with high thermal conductivities are often filled with a high volume, the cost of composites depends mainly on the price of thermal conductive fillers. Some thermal conductive minerals are considered attractive candidates. Especially aluminosilicate (AlS) and aluminum oxide (AlO), which are the major components of kaolin and other clay minerals [15]. Their advantages include a low price and good thermal conductivity. Thus, AlS- and AlO-filled epoxy composites fabricated by injection molding may offer great advantages in industrial applications. According to the Lewis-Nielsen equation [16], a high thermal conductivity of composites can also be realized as long as the volume fraction of filler is high enough [17], even though the thermal conductivities of AlS- and AlO-fillers are not as high as BN. However, compared to thermoplastic composites filled with AlS and AlO [18], the investigation of AlS- and AlO-filled epoxy composites is rarely reported. Considering the better design, cost efficiency and improved usability, it is necessary to conduct an investigation into the thermal conductivity of AlS and AlO filled epoxy composites fabricated with injection molding. 

The purpose of this study was to investigate the major factors affecting the thermal conductivity of mineral-filled thermosets composites with injection molding. The effects of filler characteristics including filler content, size, and geometry, on the thermal conductivity of the composites were investigated. In addition, the experimental data was compared with the predicted values obtained from the Lewis-Nielsen equation. The fillers and their distribution in injection molded parts were observed with scanning electron microscopy (SEM). 

## 2. Models for Predicting Thermal Conductivity of Two-Phase Polymer Composite

Many equations have been proposed to calculate the thermal conductivity of two-phase systems [16,19,20,21]. In the Lewis-Nielsen equation, the maximum volume fraction, $\phi_{\max}$, of filler was introduced to the Halpin-Tsai’s equation [16,22]. Thus, the Lewis-Nielsen equation for calculating thermal conductivity is represented as:

(1) $$\frac{\lambda_{c}}{\lambda_{m}}=\frac{1+AB\phi_{f}}{1-\psi B\phi_{f}}$$

where $=\frac{\lambda_{f}/\lambda_{m}-1}{\lambda_{f}/\lambda_{m}+A}$, $\psi =1+\frac{\left(1-\phi_{\max}\right)\phi_{f}}{\phi_{\max}{}^{2}}$, and $A=k_{E}-1$.

In these equations, $\lambda_{c},\;\lambda_{m}$ and $\lambda_{f}$ are respectively the thermal conductivity of composite, polymer matrix and filler; A is a parameter related to the generalized Einstein coefficient $k_{E}$ ($k_{E}$ = 2.5 for rigid spheres suspended in a matrix with Poisson’s ratio of 0.5), B is a constant which takes into account the relative conductivity of the two components, and $\phi_{\max}$ is the maximum packing fraction ($\phi_{\max}$ = 0.64 for randomly close packed spheres). It is well known that with a low filling degree the predicted results obtained by the Lewis-Nielsen equation fit quite well with the experimental data [17]. In this equation, the geometry of filler and the maximum packing fraction related to the particle size were taken into consideration. However, the effect of the filler orientation was not included because the randomly closed packing of fillers in polymer matrix was assumed. The fillers used in this paper have a relatively low aspect ratio, therefore, the Lewis-Nielsen equation is employed to predict the integrating thermal conductivity of composites.

## 3. Materials, Sample Fabrication and Testing Methods 

### 3.1. Materials

The functional fillers used in this paper are Silatherm 1360 EST (AlS) and Silatherm Plus 1430 EST (AlO) (Quarzwerk GmbH, Frechen, Germany), with different particle size distribution and geometry. The compositions of fillers and main physical properties are listed in Table 1. The thermal conductivity of AlS is 14 W/mK, which is lower than that of AlO (30 W/mK). Due to the high proportion of SiO_2_, AlS also shows less Mohs scale of hardness than AlO. The different classify numbers in Table 2 give an indication of gain size. The particle with classify 400 are in general, the smallest while those with classify 007 are the biggest in size. The classify 506 has a special particle size distribution, whose upper gain size is bigger than that of classify 010. Unlike AlS with cuboid geometry, the AlO particle has a spherical geometry.

Thermoset epoxy resin (EP) Epoxidur 246/1 (Raschig GmbH, Ludwigshafen, Germany) is used as the polymer matrix. It is a black powder mixture with epoxy resin, hardner, catalyst and black carbon. The composition of the mixture is not released due to business secret. The density of the mixture at room temperature is 1.23 g/cm^3^. 

### 3.2. Sample Fabrication

**Figure 1:**
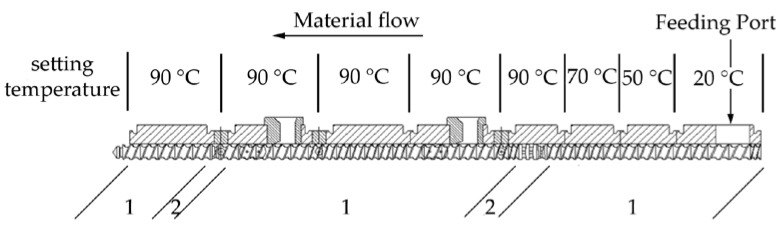
Used screw configuration and setting temperature of heating blocks for the preparation of composites (1. conveying elements; 2. kneading elements).

**Figure 2:**
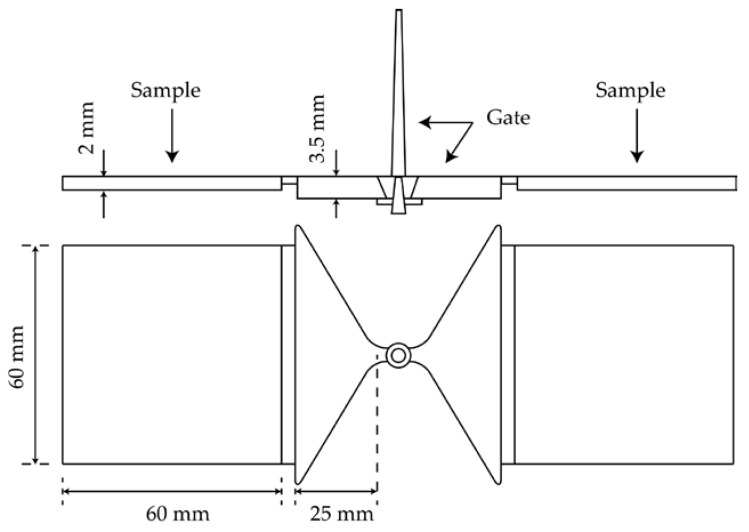
Geometry of injection molded samples.

The EP powder was premixed with fillers at a different particle size and proportion. The weight of the filler content of composites is between 65% and 80%. Compositions of the investigated composites are listed in Table 3. The mixture was fed into a twin screw extruder (ZSE 25*A* × 45*D*, KraussMaffei Berstorff GmbH, Hannover, Germany) with a screw diameter of *D* = 25 mm and a screw length of *L* = 45.5 *D*. As described in Figure 1, the screw configuration for processing thermoset composites consists of less kneading elements due to the high thermal sensitivity of epoxy resins. The maximal temperature zone was set up to 90 °C, so that EP was molten and meanwhile not able to be cured during processing. After the mixture were extruded as strings, they were cooled using a vibration chiller and further granulated with a rotation crusher in order to form suitable pellets for the injection molding process. Finally, the granules were shaped into samples through injection molding. The dimensions of the plate sample are according to EN ISO 10724-2, as shown in Figure 2. The plate sample had both a width and length of 60 mm and a thickness of 2 mm. 

### 3.3. Testing Methods

The temperature-dependent reaction kinetic of EP was studied with differential scanning calorimetry (DSC Q100, TA Instruments, New Castle, DE, USA). Samples (4–5 mg) were prepared directly in DSC aluminum pans with a lid and heated at a constant rate (20 °C/min) from 0 to 250 °C. The experiments were carried out in nitrogen at a flow rate of 50 mL/min. 

Meanwhile, the complex viscosity of EP was measured as a function of temperature with plate-plate geometry (plate radius = 25 mm) using a rheometer (AR2000, TA Instruments). The testing temperature was from 80 to 240 °C. The measurement was carried out with 5 °C/min heating rate, 0.1% strain and 1 Hz frequency. 

A scanning electron microscope (SEM) (Ultra Plus, Carl Zeiss Microscopy GmbH, Oberkochen, Germany) was used to characterize the microstructure of filler materials and their distribution in the injection molded samples. All samples were treated with a 10 nm layer of spray gold. The pictures were taken with a working distance of 11–13 mm and an acceleration voltage of 10 kV. 

The hot-disk method (TPS M1, Hotdisk AB, Göteborg, Sweden) was used to determine the thermal conductivity of samples with different fillers and filler contents. The Kapton-insulated sensor (Hotdisk AB, Göteborg, Sweden, sensor radius = 6.4 mm) of the hot-disk was sandwiched between two pieces of same samples with parallel plain surface. Because the heat from the sensor flows unidirectionally into samples, the measured thermal conductivity is integrated without directionality. In this study, five pieces of samples were measured for each composite. 

To determine the value of the aspect ratio in the Lewis-Nielsen equation, an optical camera with static image analysis (Morphologi G3s, Malvern, UK) was used to measure the distribution of the aspect ratio of AlS. The volume of sample was 3 mm^3^. The number of the counted particles was 200,000. 

## 4. Results and Discussion

### 4.1. Characterization of the Polymer Matrix and Fillers

**Figure 3:**
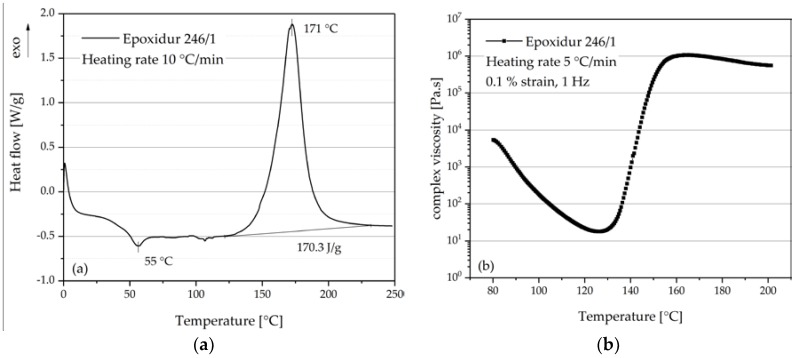
(**a**) thermal analysis of EP with differential scanning calorimetry (DSC); (**b**) complex viscosity of EP as function of temperature.

Figure 3a gives the DSC result of EP for the temperature range 0–250 °C. The melting temperature of EP is 55 °C. The curing reaction begins at about 125 °C with heating rate 10 °C/min. As the curing reaction is an exothermal process, the reaction heat is measured with 170.3 J/g. Figure 3b shows the viscosity of EP with temperature sweep from 80 to 200 °C. The viscosity of EP decreases with the increase of temperature from 80 to 128 °C. At 128 °C the viscosity reaches its minimum value of 17 Pa∙s. Then, the viscosity increases with the beginning of curing. Compared with the thermoplastic matrix, e.g., PA6 (Ultramid B3 of company BASF, Ludwigshafen, Germany) with viscosity ca. 500 Pa∙s at 250 °C and shear rate 10 s^−1^ [23], EP exhibits advantages due to its low matrix viscosity for a high volume filled composite system. Theoretically, a matrix with lower viscosity can enable composite with a higher filling degree. 

**Figure 4:**
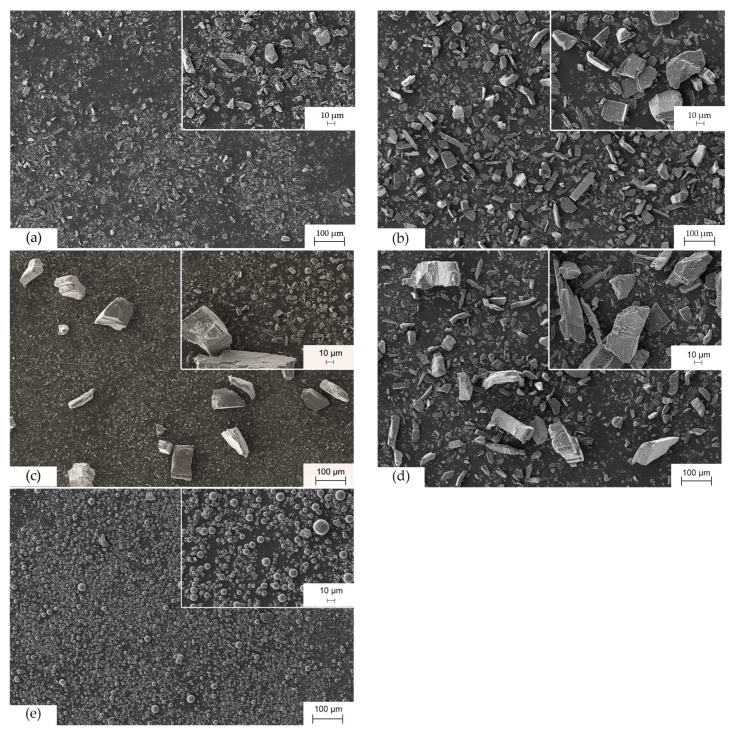
Microstructure of fillers photographed by SEM. (**a**) Silatherm 1360-400; (**b**) Silatherm 1360-010; (**c**) Silatherm 1360-506; (**d**) Silatherm 1360-007; (**e**) Silatherm Plus 1432-400.

Figure 4 shows the microstructure of fillers used in this paper. Figure 4a–d are the distribution of AlS with different particle size. AlS particles generally have a cuboid geometry. Whereas, AlO particles are rigid spheres. 

**Figure 5:**
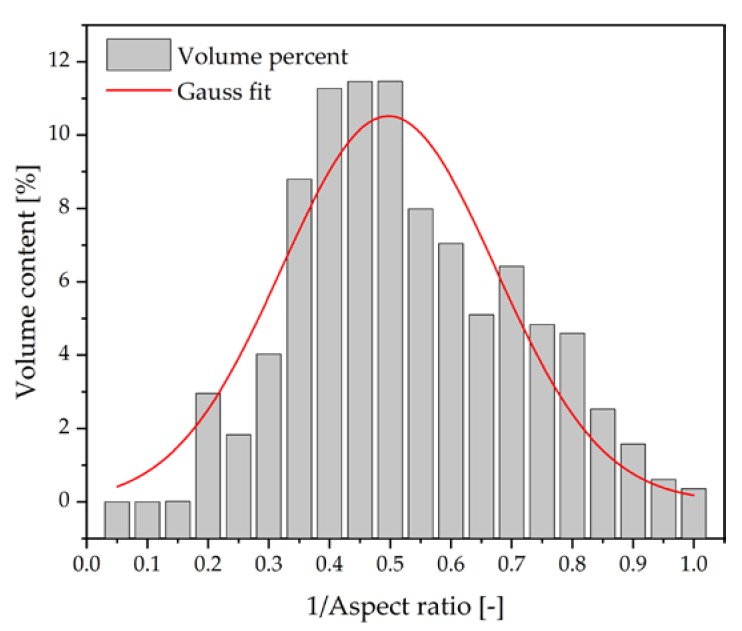
Measured distribution of aspect ratio of Silatherm 1360-007.

Figure 5 shows the volume content versus the inverse aspect ratio of Silatherm 1360-007. It can be seen that the inverse aspect ratio follows Gauss distribution. The mean value of the inverse aspect ratio of Silatherm 1360-007 was found to be 0.497, which was obtained through least squares regression. 

### 4.2. Thermal Coductivity of Composites

**Figure 6:**
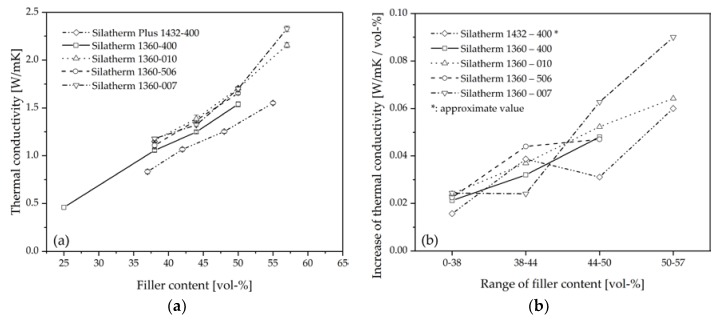
(**a**) Measured thermal conductivity of composites with different filler and filler content; (**b**) Increase of thermal conductivity with 1 vol % more filler in different filler content range.

Figure 6a shows the effects of different fillers and filler contents on the thermal conductivity of composites fabricated by injection molding. The filler content is defined as the volume fraction of fillers. As can be seen, the thermal conductivity of composites increases with an increasing filler content regardless of filler size and type. It indicates that the increase of thermal conductive filler content in composites is always an effective approach to improve the thermal conductivity. 

On the other hand, the filler geometry is an important factor in the thermal conductivity of composites. The composites composed of AlO (Silatherm 1432-400) with higher thermal conductivity (30 W/mK) exhibit lower thermal conductivity than those composed of AlS (Silatherm 1360-400, 14 W/mK), even though the same filler content and size classify were used. The spherical geometry of AlO results in the lower thermal conductivity of composites than the cuboid geometry of AlS. Similar results can be found in Amersoede’s work [14]. In his dissertation, PA6 was used as the polymer matrix. Coppers with different geometries were introduced as the filler. The experimental results showed that the spherical filler delivers the lowest contribution to the increase in the thermal conductivity. 

In addition, composites composed of fillers with a relatively bigger particle size (AlS, Silatherm 1360-007, *D*50 = 28 µm) show higher thermal conductivity than those with a smaller particle size (AlS, Silatherm 1360-400, *D*50 = 5 µm). The result shows a tendency for the higher thermal conductivity composites to be achieved when the filler size is larger. Especially when the filling degree is over 50 vol %. 

Figure 6b shows the relative increase in thermal conductivity of composites for every 1 vol % filler in a different filler content range. Because the composites with Silatherm 1432-400 have slightly different volume contents from those with Silatherm 1360-series, the value in Figure 6b for Silatherm 1432-400 was approximated, so that it can be compared with other fillers. The thermal conductivity of a commercial EP resin is about 0.25 W/mK [8]. In the filling range of 0–38 vol %, if 1 vol % more filler is used, the thermal conductivity of composites increases only about 0.02 W/mK. However, the contribution of 1 vol % filler to the thermal conductivity improves with the increasing of total filler content. In the filling range of 50–57 vol %, the thermal conductivity increases more than 0.058 W/mK with 1 vol % more filler in the composite. That is almost 3 times more than in the low filling range (0–38 vol %), which means fillers in high filled composites are more efficient in improving the thermal conductivity. 

**Figure 7:**
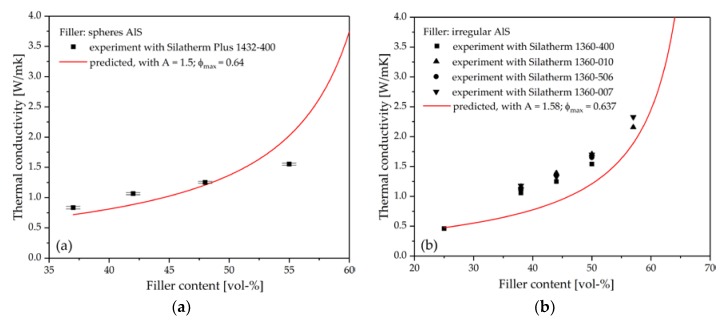
(**a**) Comparison of the measured and predicted thermal conductivity using the Lewis-Nielsen equation for composites with AlO (Silatherm 1432-400) as filler; (**b**) Comparison of measured and predicted thermal conductivity using the Lewis-Nielsen equation (*A* = 1.58 and $\phi_{\max}$ = 0.637).

The predicted results of thermal conductivity obtained from the Lewis-Nielsen equation were compared with the experimental data. Because the filler Silatherm Plus 1432-400 is a rigid sphere as shown in Figure 4e, the filler geometry-dependent coefficient A in the equation was defined as 1.5 and the maximum packing fraction $\phi_{\max}$ of 0.64 was used [16]. Figure 7a shows that the predicted results fit the experimental data well when the filler content is less than 50 vol %. The thermal conductivity increases with an increasing filler content. It is explained by Burger et al. [24] that more fillers in the composite results in fewer thermal-resistant interfaces, which then causes higher thermal conductivity. While the filler content is over 50 vol %, the predicted results are much higher than the experimental data. The same phenomena can be found in many publications [16,17,24,25]. A possible explanation for this is that the potential agglomeration of fillers is bigger for a high volume filled composite. This introduces voids into the composite and reduces the thermal conductivity.

To calculate the thermal conductivity of composites composed of AlS (Silatherm 1360-serie), the value of the filler geometry-dependent coefficient A and the maximum packing fraction, $\phi_{\max}$, are needed. As shown in Figure 4a–d, AlS (Silatherm 1360-serie) has an irregular geometry, thus an exact value of *A* is not available. According to the results in Figure 5, an aspect ratio of ca. 2 for AlS is evaluated. For irregular random closed particles, Bigg et al. [25] recommended the value of coefficient *A* as 1.58 and the maximum packing fraction, $\phi_{\max}$, as 0.637. Thus, the experimental results of thermal conductivity of composites composed of AlS and the predicted results according to the Lewis-Nielsen equation are shown in Figure 7. As seen in Figure 7a, the Lewis-Nielsen equation can fit the experimental results well for composites composed of spheres AlO. On the contrary, the predicted results for composites composed of irregular AlS are generally smaller than the experimental values. This could be due to the characteristic of the irregular shape of particles. It can be understood that the Lewis-Nielsen equation assumed the fillers were packed randomly in the polymer matrix without external forces. During the injection molding process, fillers with irregular geometry could orient in the flow direction under share forces. Heinle [26] illustrated that if an orientation of fillers was built, the integrated thermal conductivity can be improved.

### 4.3. Distribution of Fillers in Injection Moled Samples

**Figure 8:**
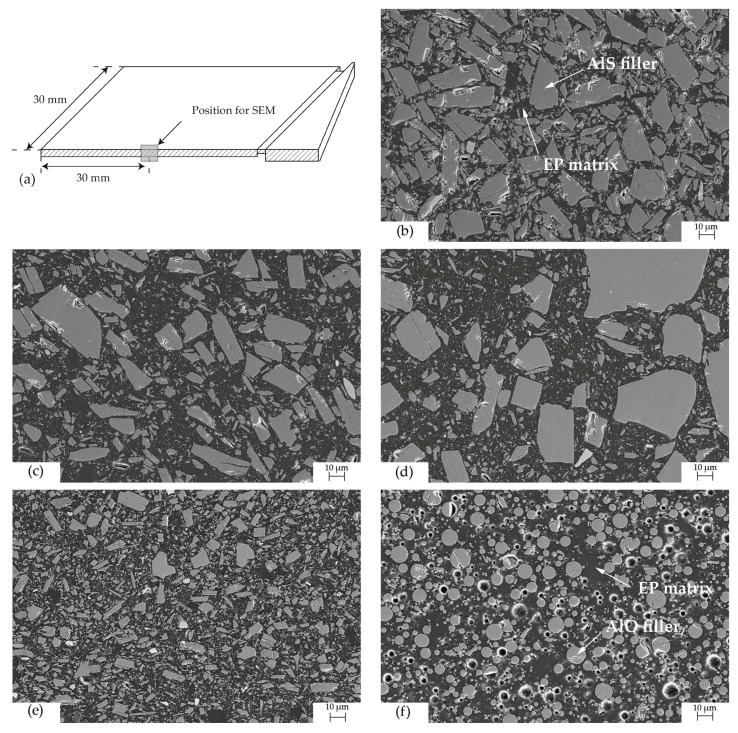
Microstructure of fillers in injection molded parts. (**a**) Position for SEM. (**b**) EP with 57 vol % AlS (Silatherm 1360-010); (**c**) EP with 38 vol % AlS (Silatherm 1360-010); (**d**) EP with 38 vol % AlS (Silatherm 1360-007); (**e**) EP with 38 vol % AlS (Silatherm 1360-400); (**f**) EP with 37 vol % AlO (Silatherm Plus 1432-400).

The distribution of fillers in injection molded samples was observed with SEM. Figure 8b–e gives the distribution of AlS with different particle sizes and filler content in the EP matrix. As the filler content decreases from 57 to 38 vol %, thermal-resistant interfaces increase significantly due to more EP in the composite (Figure 8b,c). Therefore, the increase of thermal conductivity with 1 vol % more filler for high filled composites is more significant than that for low filled composites. Composites with bigger filler particle size have more chance to build a path with less thermal-resistant interfaces through a small region (Figure 8d,e). With equal filler content, the filler with sphere geometry has more interfaces than that with cuboid geometry (Figure 8e,f). Thus, the thermal conductivity of composites with cuboid fillers is higher than that with sphere fillers. 

## 5. Conclusions

In this work, AlS- and AlO-filled EP composites were first fabricated with TSE and then shaped into plate samples using injection molding. The results obtained from the investigation of thermal conductive AlS- and AlO-filled EP composites allowed us to draw the following conclusions:

1. For AlS and AlO filler, the thermal conductivity increases with an increasing filler content. It confirms that the increase of the content of thermal conductive filler is an important approach to improve the thermal conductivity of composites. When the filler content of composites is over 50 vol %, the increasing of fillers is more effective than low filled composites;
2. Smaller fillers cannot improve the thermal conductivity as well as larger fillers can. Thus, fillers with bigger particle size should be considered preferable as raw material for composites with a high thermal conductivity;
3. Fillers with sphere geometry are not as effective as those with cuboid geometry to improve the thermal conductivity. This is attributed to the smaller surface area of sphere fillers and more thermal-resistant interfaces;
4. The Lewis-Nielsen equation is suitable to predict the thermal conductivity of a two-phase system with spheres filler content within 50 vol %. For irregular fillers, the predicted results obtained are smaller than the experimental values.

## Figures and Tables

**Table 1:** Composition and some physical properties of AlS and AlO (information from manufacturer).

Product	Typical chemical analysis (Weight %)					Density (g/cm^3^)	Thermal conductivity (W/mK)	Mohs hardness (-)
	Al_2_O_3_	SiO_2_	TiO_2_	Fe_2_O_3_	Rests			
Silatherm 1360	55	43	1	0.5	0.5	3.65	14	5
Silatherm Plus 1432	99	-	-	-	1	4	30	9

**Table 2:** Particle size distribution of fillers (information from manufacture).

Product		Silatherm 1360 EST *				Silatherm plus 1432 EST *
Classify		007	506	010	400	−400
Upper gain size	D90% in µm	94	129	22	12	16
Average gain size	D50% in µm	28	10	9	5	5
Lower gain size	D10% in µm	2	1	1	1	1
Particle geometry		Cuboid				Spherical

* EST: treated with epoxysilane.

**Table 3:** List of used fillers and their content in composites.

Filler	Classify of particle size	Filler content	
		(Weight %)	(Volume %)
AlS (Silatherm 1360)	007	65, 70, 75, 80	38, 44, 50, 57
AlS (Silatherm 1360)	506	65, 70, 75	38, 44, 50
AlS (Silatherm 1360)	010	65, 70, 75, 80	38, 44, 50, 57
AlS (Silatherm 1360)	400	50, 65, 70, 75	25, 38, 44, 50
AlO (Silahterm Plus 1432)	400	65, 70, 75, 80	37, 42, 48, 55
